# Case Report: Seaweed Bezoar Masquerading as a Malignant Obstruction

**DOI:** 10.1155/2018/3829271

**Published:** 2018-10-28

**Authors:** E. C. Abboud, B. Babic

**Affiliations:** New York-Presbyterian/Queens, Weill Cornell Medical College, Flushing, NY 11355, USA

## Abstract

Bezoars represent a rare cause of small bowel obstruction (SBO). Nonoperative management of bezoars includes use of endoscopy with mechanical or chemical dissolution methods. When obstruction persists, surgical intervention becomes necessary. Here, we present the case of an Asian woman with a SBO secondary to a phytobezoar masquerading as a malignancy. She presented with two days of acute-on-chronic abdominal pain that started after eating seaweed. Initial computed tomography (CT) scan showed a SBO with a jejunal transition point and ill-defined liver lesions, worrisome for a malignant obstruction with liver metastases. Further imaging, however, showed the resolution of these artifacts. Subsequent laparotomy revealed a small bowel loop with copious obstructing seaweed. A distal stricture was palpated, and the involved segment was resected. Intraoperative liver ultrasound was negative, and final pathology revealed benign small intestine with a mild stricture. Given the rarity of bezoar-related obstructions, the diagnosis is often delayed particularly when confounding factors exist such as our patient's concomitant hepatic findings. Contrast-enhanced CT has a high sensitivity but a lower specificity in identifying bezoars. A high index of suspicion is therefore necessary especially when managing higher risk patients.

## 1. Introduction

Small bowel obstructions (SBOs) are common surgical encounters. There are a large variety of causes with >50% caused by adhesions. Bezoars are masses of ingested undigested materials that accumulate in the gastrointestinal lumen [1, 2]. They represent a rare cause of SBO accounting for 0.4–4% of cases [1]. The most common types are phytobezoars composed of plant fibers, most commonly caused by the persimmon fruit [2, 3]. Other types include lactobezoars and trichobezoars, caused by the aggregation of dairy products and hair, respectively [4, 5]. At-risk patients include those with previous gastric surgery, excessive consumption of some fruits and vegetables, and mental disorders [6]. Here, we present the rare case of a patient who suffered a SBO secondary to a seaweed phytobezoar masquerading as a malignancy.

## 2. Case Presentation

**Figure 1:**
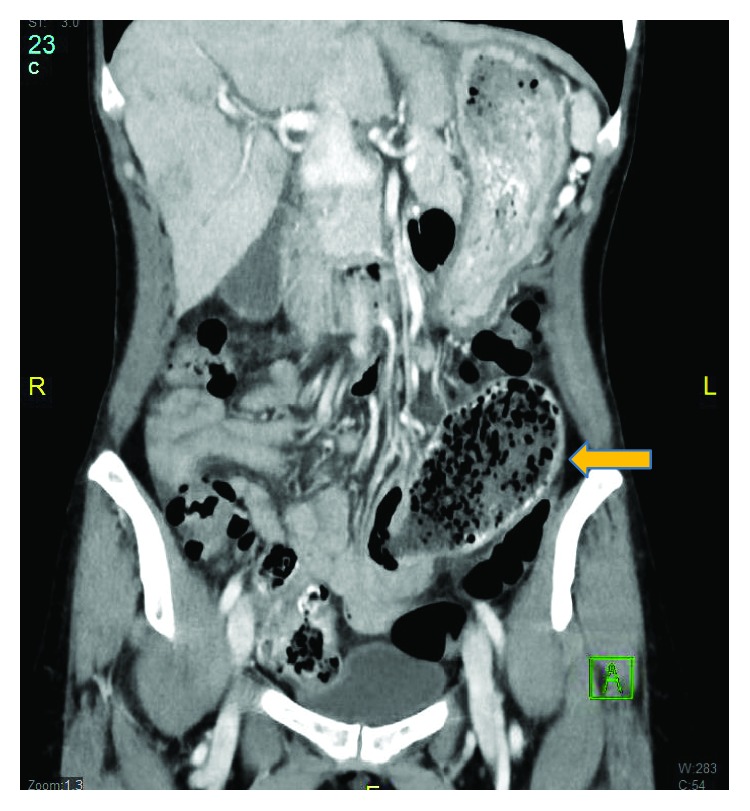
CT scan of the abdomen and pelvis showing a dilated jejunum and transition point.

**Figure 2:**
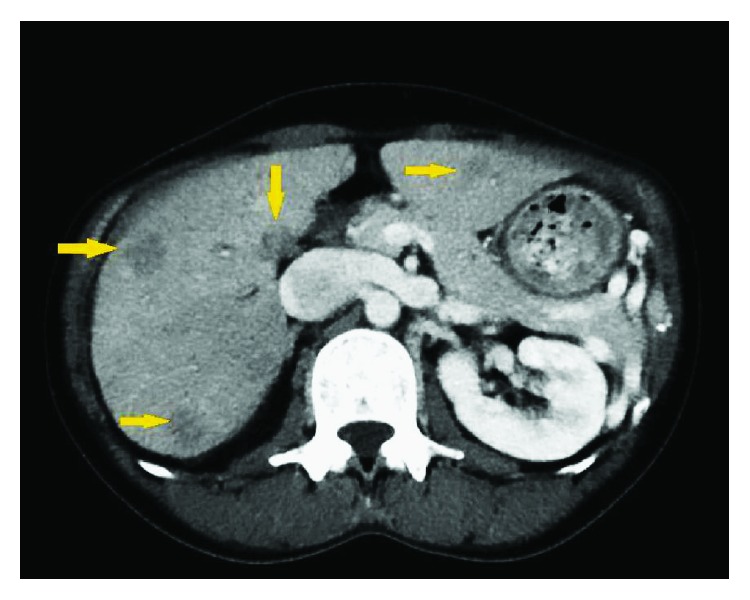
CT scan showing some of the multiple ill-defined liver lesions concerning for metastatic disease in setting of a primary SBO.

**Figure 3:**
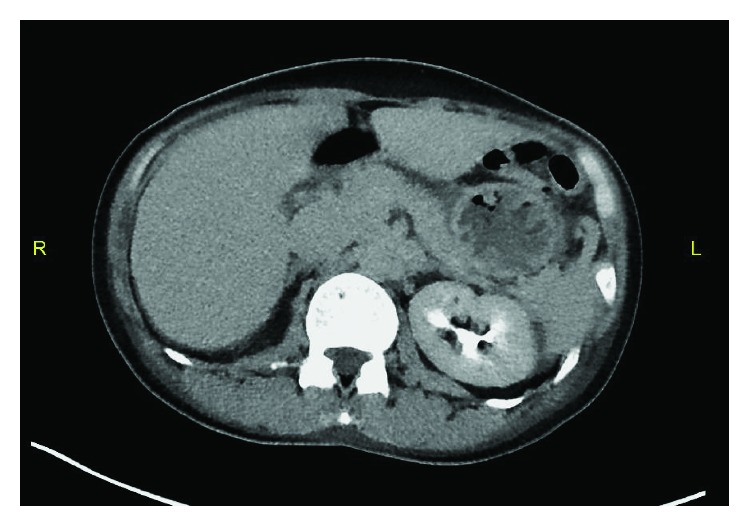
Repeat CT scan showing the absence of the previous liver artifacts.

**Figure 4:**
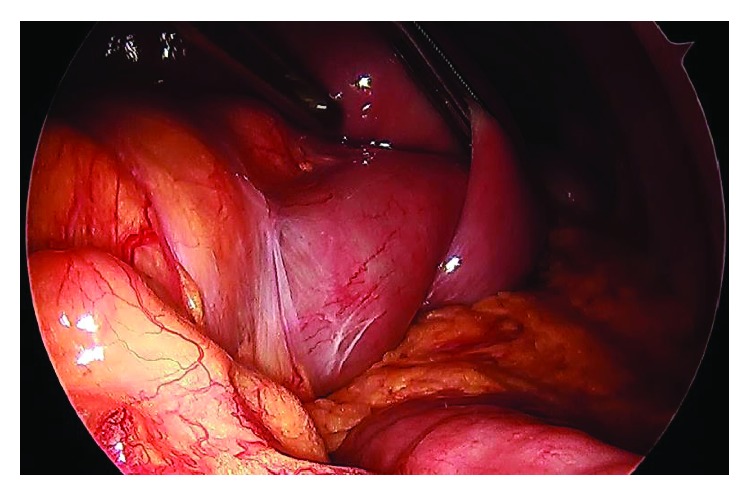
Intraoperative laparoscopic view of dilated jejunum.

**Figure 5:**
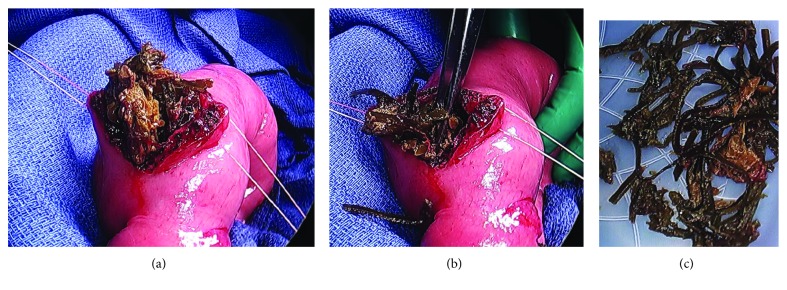
(a, b) Intraoperative view of proximal jejunum after enterotomy showing phytobezoar. (c) Copious amounts of evacuated seaweed.

A 51-year-old Asian lady with no surgical history presented to the emergency room with two days of emesis and abdominal pain. She had been experiencing intermittent abdominal pain for a year, but her symptoms acutely worsened after eating a seaweed salad. Initial computed tomography (CT) scan showed a SBO with transition point in the jejunum (Figure 1) and multiple nonspecific ill-defined liver lesions (Figure 2), worrisome for a malignant obstruction with liver metastases. Tumor biomarkers alpha-fetoprotein (AFP) and carcinoembryonic antigen (CEA) were checked and found to be negative. Repeat CT scan at the time of a planned CT-guided liver biopsy showed the absence of the previous liver lesions, now known to be artifactual (Figure 3). The SBO persisted, so the decision was made to intervene surgically. Diagnostic laparoscopy showed a dilated proximal jejunum with no adhesions (Figure 4). The bowel was edematous, friable, and difficult to maneuver, so we converted to a small laparotomy. The involved segment's consistency was soft and suspicious for a bezoar. An enterotomy was made and revealed a copious amount of obstructing seaweed (Figure 5). A distal stricture was palpated, and the involved segment was resected. Intraoperative liver ultrasound was negative. There were no complications, and the patient was discharged home four days later. Final pathology revealed benign small intestine with a mild benign stricture.

## 3. Discussion

Obstructive bezoars can be managed in several different ways. Nonoperative methods include the use of endoscopy with mechanical or chemical dissolution. When surgical intervention is needed, both laparoscopic and open approaches have shown similar outcomes [1]. Our patient did not improve without surgery, which was also required to definitively rule out a malignancy. Given the rarity of bezoar-related SBOs, the diagnosis can be challenging and is often delayed. This is especially true when confounded by other findings such as this patient's concomitant hepatic lesions, artifacts, which were likely caused by transient attenuation differences at various phases of normal perfusion. Contrast-enhanced CT has been shown to be 90% sensitive but only 57% specific in identifying bezoars [1]. A high index of suspicion is therefore necessary especially when managing at-risk patient populations [6].
